# Abundance and Distribution of Malaria Vectors in Various Aquatic Habitats and Land Use Types in Kakamega County, Highlands of Western Kenya

**DOI:** 10.4314/ejhs.v31i2.7

**Published:** 2021-03

**Authors:** Kitungulu Nicholas, Guyah Bernard, Ndenga Bryson, Kipcho Mukabane, Mark Kilongosi, Stephen Ayuya, David Hughes Mulama

**Affiliations:** 1 School of Public Health & Community Development, Maseno University, Kenya; 2 School of Natural Sciences, Biological Sciences Department, Masinde Muliro University of Science & Technology, Kenya; 3 Centre for Global Health Research, Kenya Medical Research Institute, Kisumu, Kenya; 4 School of Health Sciences, Kirinyaga University, Kenya

**Keywords:** Anopheles larvae, aquatic habitat, land use type, malaria vector, malaria transmission

## Abstract

**Background:**

Management of malaria transmission relies heavily on vector control. Implementation and sustenance of effective control measures require regular monitoring of malaria vector occurrences, species abundance and distribution. The study assessed mosquito larval species composition, distribution and productivity in Kakamega County, western Kenya.

**Methods:**

A cross-sectional survey of Anopheline larvae was conducted in various aquatic habitats and land use types in Kakamega County, highlands of western Kenya between the month of March and June 2019.

**Results:**

One thousand, five hundred and seventy six aquatic habitats were sampled in various land use types. The mean densities of An. gambiae s.l (46.2), An. funestus (5.3), An. coustani (1.7), An. implexus (0.13) and An. squamosus (2.0) were observed in fish ponds, burrow pits, drainage ditches, and tire tracks, respectively. High mean densities of An. gambiae s.l was reported in farmland (20.4) while high mean abundance of An. funestus s.l (8.2) and An. coustani s.l (4.0) were observed in artificial forests.

**Conclusion:**

The study revealed that the productivity of anopheles larvae varied across various habitat types and land use types. Therefore, treatment of potential breeding sites should be considered as an additional strategy for malaria vector control in Kakamega County, western Kenya.

## Introduction

Malaria infections have been re-emerging in highlands of Western Kenya (1). Kakamega County is among the counties characterized under the Lake endemic region with high prevalence (2). Even though highland regions have continuously been considered unsuitable for malaria vector breeding, malaria transmission often occurs as epidemics in these areas (3). Infection of malaria has been intensely associated with distribution, abundance and occurrence of anopheline mosquitoes (4). Environmental, altitudinal and ecological settings affect the vector densities (3,5).

Mosquito life cycle consists of four stages occurring in aquatic and terrestrial habitats (6). Larvae are highly limited to aquatic habitats with slight possibilities of evading prevention methods unlike free-flying adult mosquitoes hence making larviciding an effective control approach. Integrating larval with adult control methods have shown to significantly decrease mosquito populations (7). Adult and larval malaria vector monitoring plays a key role in provision of information on anopheline mosquito species and distribution for adoption of efficient prevention and control strategies in malaria transmission.

Malaria vector abundance and distribution is vital in determining disease transmission and prevalence (4,8). Studies on vectorial systems of anopheles species variations have revealed to be beneficial in characterization, monitoring and control of mosquito vectors (9,10). The main malaria vectors in Western Kenyan are known to be *Anopheles gambiae* s.s (sensu stricto), *Anopheles arabiensis* and *Anopheles funestus* s.l (sensu lato) (11). Mosquito control methods particularly indoor residual spraying (IRS) and insecticide treated bed-nets (ITNs) are key in malaria prevention (12). With increasing number of malaria infections in the highlands of Western Kenya (13), an urgent need for baseline survey on malaria vector distribution and abundance was necessary to generate information for proper elimination and control of the disease.

Land use type, aquatic habitat type and land use changes have shown to influence malaria vector productivity that supports stable malaria transmission in highlands of Western Kenya (14–18). Land use change and alteration of various aquatic water bodies has been caused by increasing population pressure which has led to natural swamps modification, deforestation and change in farming practices (15,17). These activities have resulted in open water bodies that provide conducive environment for mosquito breeding and vector succession which may contribute to increasing malaria infections (15,17). Previous studies show that *Anopheles gambiae* s.l equally breeds in different aquatic habitats (4,15). Interestingly, habitat vector distribution shows geographic and species diversity over time (19). However, there is no information on vector distribution in relation to land use and aquatic habitat in Kakamega County. Therefore, the present study investigated abundance and distribution of malaria vectors in various aquatic habitats in Kakamega County, highlands of Western Kenya.

## Materials and Methods

**Study area:** The study was conducted in Kakamega County, highlands of Western Kenya (Ikolomani; E:00.16556, N:034.73194, Mumias East; E:00.34120, N:034.54727, Lurambi; E:00.31806, N:034.75222 and Malava; E:00.32957, N:034.74701). The county is situated in Western Kenya, a few kilometers away from the Equator; its geographical coordinates are 0° 17′ 0″ North, 34° 45′ 0″ East with an average elevation of 1,535 metres. The area contains Kakamega and Malava forests a remnant of a rainforest that once stretched west through Uganda (“Geography/Topography | Republic of Kenya | The Embassy of the Republic of Kenya,”). The county experiences two peaks of rains with an average annual rainfall of 1921 mm. The first peak of rains occurs between March and July having an average monthly rainfall of 205 mm. The dry period occurs between December to February with an average of about 79.5 mm. Temperature within the study area ranges between annual maximum of 21.4°C and minimum of 19.3°C with a daily average temperature of 20.5°C (22). The rainfall patterns experienced in these areas determine greatly the malaria vector productivity and occurrence hence transmission of malaria infection. A two weeks lag period is the duration through which the mosquito aquatic life cycle and parasite development undergoes before transmission can be initiated. Sugarcane farming is the main agricultural activity in Kakamega County. Besides sugarcane farming, residents also practice food crop farming and rearing of animals. River/stream basins have been reclaimed for growing a wide range of food crops. Other socio-economic activities the residents engage in are small scale trade and bodaboda.

**Study design**: A cross-sectional larval survey was done between March to June 2019. All aquatic habitats present during the period of survey were sampled for presence of *Anopheles* larvae and any other mosquito.

**Mosquito sampling**: Anopheles mosquito larvae were sampled in aquatic habitats using a standard 350 milliliter dipper between 0830Hrs to 1130Hrs and GPS co-ordinates recorded. Three to ten dips were made depending on the size of the habitat. All aquatic habitats showing presence of immature malaria vectors were sampled and identified morphologically using the key developed by Gillies (23) and abundance scored.

**Characteristics of various aquatic habitat types and Land use types surveyed:**

Characteristics of various aquatic habitats were described as: (i) drainage ditches; this were open narrow drainages created to channel water to main stream/river; (ii) river/stream these were edges of a river or streams; (iii) natural swamps were water-logged sections of land with reed and tall grasses; (iv) Cultivated swamps were water-logged sections of land for cultivation or being cultivated; (v) burrow pits were where land surface had been dug to do a specific purpose either hold water for construction work or brick making; (vi) puddles were a small accumulation of water on a surface after rainfall; (vii) gold mines; were places where gold ore had been removed from the ground surface and left open; (viii) footprints; were a mark left behind of a foot that a person or animal created on land/soil surface; (ix) fish ponds were an artificial structure used for fish farming; (x) rock pools were a pool of water on rocks or rock caves and lastly; (xi) tire tracks these were impression left by tires on the surface onto which vehicles or tracks drove. While characteristics of land use types were described as: (i) farmland were land under cultivation; (ii) pasture land were described as land covered with grass suitable for grazing of livestock; (iii) artificial forest were forests that comprised nonnative and or native tree species and differed from natural forests in structure, composition and intensity of management; (iv) bushes were described as a large uncleared sparsely settled area with plants growing with many small stems that are less than 2 meters high; (v) swamp land were characterized as low land that was seasonally flooded or land that was saturated with water; (vi) roadsides were strips of land along roads or sides of the roads and lastly (vii) river/stream land were characterized as the area of land where water flew into stream or river proximate to its sources ‘riparian’.

**Data management and analysis**: Statistical analyses were performed using SPSS version 19.0 for Windows (IBM SPSS Statistics for Windows, Version 19.0. Armonk, NY: IBM Corp). For abundance and distribution, analysis of variance (ANOVA) tests were used. Tukey-honestly significant difference (HSD) tests were used for multiple comparison of mean abundance. Alpha level of 5% was accepted as statistically significant.

**Ethics statement**: The study was approved by the Maseno University Ethical Review Committee (MUERC) under the scientific steering committee (MUERC/0061/18). Written informed consent was obtained from farm owners in the study area. This study did not involve endangered or protected species.

## Results

**Aquatic habitat and land use characteristics in the four study sites of Kakamega County:** Characteristics are summarized in Table 1. For aquatic habitats, proportions of drainage ditches was significantly different across the four study sites (P<0.0001) such that the proportion of drainage ditches was higher in Malava (30.2%) compared to Mumias east (27.0%), Lurambi (22.0%) and Ikolomani (20.7%). There was no statistical difference in proportion of footprints across the study areas (P=0.166). There was significant difference in proportions of burrow pits across the study areas (P=0.020). However, there was no significant difference in proportions between Ikolomani (30.3%) vs. Lurambi (31.8%) and Mumias east (22.4%). The same to Mumias east (22.4%) and Malava (15.4%). No analysis of variance was computed on gold mines, cultivated swamps, tire tracks, rock pools, fish ponds and puddles due to lack of counts in some of the aquatic habitats in some areas. On land use type, there was statistical difference on farmland proportions (P<0.0001) across the study areas. Although there was no significant between Malava (28.9%) and Mumias east (27.9%). No analysis of variance was computed on pasture land, swamp land, river/stream fringes, artificial forests, bushes and roadsides due to lack of counts in some areas.

**Table 1:** Aquatic habitat and land use characteristics in the four study sites of Kakamega County, highlands of western Kenya

Aquatic habitat & Land use characteristics	Study area				

	Ikolomani n (%)	Lurambi n (%)	Malava n (%)	Mumias east n (%)	P value
**Aquatic habitat**					
Drainage ditches	224 (20.7)^a^	238 (22.0)^a^	327 (30.2)	292 (27.0)	**<0.0001**
Foot prints	14 (32.6)	13 (30.2)	4 (9.3)	12(27.9)	**0.166**
Gold mine	90 (61.2)	57 (38.8)	0 (0.0)	0 (0.0)	-
Burrow pits	61 (30.3)^a^	64 (31.8)^a^	31 (15.4)^b^	45 (22.4)^a^, ^b^	**0.020**
Cultivated swamp	20 (64.5)	8 (25.8)	2 (6.5)	4 (3.2)	-
Tire tracks	0 (0.0)	2 (50.0)	0 (0.0)	2 (50.0)	-
Rock pools	2 (16.7)	8 (66.7)	2 (16.7)	0 (0.0)	-
Fish ponds	10 (50.0)	3 (15.0)	3 (15.0)	4 (20.0)	-
Puddles	0 (0.0)	26 (96.3)	1 (3.7)	0 (0.0)	-
**Land use**					
Farmland	241 (19.2)	301 (24.0)	363 (28.9)^a^	351 (27.9)^a^	**<0.0001**
Pasture land	100 (58.8)	65 (38.2)	2 (1.2)	3 (1.8)	-
Swamp land	35 (70.0)	15 (30.0)	0 (0.0)	0 (0.0)	-
River/stream	25 (54.3)	17 (37.0)	4 (8.7)	0 (0.0)	-
Artificial forest	18 (52.9)	16 (47.1)	0 (0.0)	0 (0.0)	-
Bushes	3 (100.0)	0 (0.0)	0 (0.0)	0 (0.0)	-
Roadsides	0 (0.0)	5 (62.5)	1 (12.5)	2 (25.0)	-

a P<0.05

b P<0.05

n; number of habitats; mean of various vector species is presented alongside the confidence intervals; numbers in parentheses are 95% confidence intervals. P value = significant test at alpha level of 0.05; The table describes the mean of various proportions and mean of various aquatic habitats and land use type per study site and multiple comparisons across the study sites; letters following numbers indicate the results of Tukey-Kramer honestly significant difference tests. Letters in a row show similar significant. -; no analysis of variance since some aquatic habitats land use type had no anopheline mosquito count. P values are in bold

**Malaria larval vectors abundance and distribution in the four study areas**: Abundance and distribution of malaria vectors species and multiple comparison data is presented in Table 2. Mean abundance of *An. gambiae* s.l was significantly different across the four study sites (P<0.0001) such that mean density was higher in Malava compared to Lurambi (25.0 vs. 10.2; P< 0.0001) and Ikolomani (25.0 vs. 18.5; P= 0.138 ), but similar to Mumias east (25.0 vs. 22.7; P= 0.877). In addition, the mean density of *An. gambiae* s.l was higher in Mumias east compared to Lurambi (22.7 vs. 10.2; P< 0.0001) while mean density in Ikolomani was higher than Lurambi (18.5 vs. 10.2; P= 0.024). Mean abundance of *An. funestus* s.l significantly differed across the study sites such that mean density in Mumias east was higher than Malava (4.6 vs. 0.4; P<0.0001) while that of Ikolomani was higher than Malava (2.8 vs. 0.4; P= 0.007). Furthermore, on post Tukey-HSD tests analysis the mean density in Lurambi was higher than that of Ikolomani (5.5 vs. 2.8; P= 0.002) and Malava (5.5 vs. 0.4; P< 0.0001). Mean abundance of An. coustani s.l significantly varied across the study sites on post Tukey-HSD tests analysis such that mean density of Malava was higher compared to Mumias east (2.7 vs. 1.0; P< 0.0001 ), Lurambi (2.7 vs. 1.0; P< 0.0001) and Ikolomani (2.7 vs. 0.8; P< 0.0001).

**Table 2:** Malaria larvae vector abundance and distribution in selected study sites

Malaria vector species	STUDY SITES				*P* value

	Ikolomani (n = 422)	Lurambi (n = 419)	Malava (n = 370)	Mumias east (n = 356)	
*An. gambiae* s.l	18.5 (14.452 – 22.600) a, b	10.2 (7.459 – 13.023) a, c	25.0 (20.011 – 30.027) b, c, d	22.7 (17.672 – 27.648) b, c, d	*P*<0.0001
*An. funestus* s.l	2.8 (2.080 – 3.590) a, b	5.5 (3.930 – 7.110) a, d	0.4 (0.130 – 0.570) a, c	4.6 (3.510 – 5780) b, c, d	*P*<0.001
*An. coustani* s.l	0.8 (0.570 – 1.100^b^, ^a^	1.0 (0.731 – 1.359) b, c, d	2.7 (1.942 – 3.512) a, c	1.0 (0.653 – 1.487) b, d, c	*P*<0.001
*An. implexus*	0.0 (0.000 – 0.000)	0.0 (0.000 – 0.000)	0.0 (0.000 – 0.000)	0.2 (0.028 – 0.365)	–
*An. squamosus*	0.0 (0.000 – 0.000)	0.0 (0.000 – 0.000)	0.01 (0.005 – 0.016)	0.2 (0.046 – 0.308)	–

a P<0.05

b P>0.05

c P<0.05

d P>0.05

n, number of habitats; mean of various vector species is presented alongside the confidence intervals; numbers in parentheses are 95% confidence intervals. P value = significant test at alpha level of 0.05; *An. Anopheles* s.l, sensu lato. The table describes the mean of various malaria vector species per study site and multiple comparisons across the study sites; letters following numbers indicate the results of Tukey-Kramer honestly significant difference tests. Letters in a row show similar significant. -; no analysis of variance since some land use type had no anopheline mosquito count P values are in bold

**Malaria larval vectors abundance and distribution in various aquatic habitats across the study areas**: Abundance and distribution of malaria larval vector species in various aquatic habitats is presented in Table 3. Higher mean abundance of *An. gambiae* s.l was reported in fish ponds (46.2)compared to gold mines (29.8) and drainage ditches (18.6) while low abundance was observed in tire tracks (1.0) (P=0.001). High mean abundance of *An. funestus* s.l was observed in the burrow prints (5.3), fish ponds (4.6) and gold mine (4.1) while habitats such as tire tracks, rock pools and puddles did not record any presents of *An. funestus* s.l larvae. The highest mean abundance of *An. coustani* s.l was recorded in the drainage ditches (1.7). The highest mean abundance of *An. implexus* was reported in the burrow pits (0.13). High mean abundance of *Anopheles squamosus* was only recorded in the Tire track (2.0).

**Table 3:** Malaria vector abundance and distribution in various aquatic habitats a cross Kakamega County, highlands of western Kenya

Aquatic habitat type	Anopheline larval species				

	*An. gambiae* s.l	*An. Funestus* s.l	*An. Coustani* s.l	*An. implexus*	*An.* *squamosus*
Drainage ditches	18.6 (16.1 – 21.00)	3.20 (2.54 – 3.85)	1.66 (1.34 – 1.98)	0.03 (0.01 – 0.07)	0.00 (0.00 – 0.00)
Foot prints	29.80 (19.6 – 39.90)	0.91 (0.11 – 1.70)	1.07 (0.86 – 3.00)	0.00 (0.00 – 0.00)	0.00 (0.00 – 0.00)
Gold mine	29.80 (19.6 – 39.90)	4.10 (2.59 – 5.62)	0.80 (0.35 – 1.25)	0.00 (0.00 – 0.00)	0.00 (0.00 – 0.00)
Burrow pits	15.8 (11.30 – 20.40)	5.27 (3.25 – 7.29)	0.77 (0.44 – 1.11)	0.13 (0.13 – 040)	0.00 (0.00 – 0.00)
Cultivated swamp	4.6 (2.20 – 11.40) b	1.32 (0.36 – 3.01)	0.97 (0.52 – 2.46)	0.00 (0.00 – 0.00)	0.00 (0.00 – 0.00)
Tire tracks	1.0 (1.80 – 3.80)	0.00 (0.00 – 0.00)	0.00 (0.00 – 0.00)	0.00 (0.00 – 0.00)	2.00 (3.55 – 7.55)
Rock pools	14.2 (13.30 – 41.60)	0.00 (0.00 – 0.00)	0.00 (0.00 – 0.00)	0.00 (0.00 – 0.00)	0.00 (0.00 – 0.00)
Fish ponds	46.2 (9.50 – 82.90) a, b	4.60 (0.37 – 8.83)	1.60 (0.07 – 3.27)	0.00 (0.00 – 0.00)	0.00 (0.00 – 0.00)
Puddles	3.3 (0.50 – 7.30) a	0.00 (0.00 – 0.00)	0.41 (0.16 – 0.97)	0.00 (0.00 – 0.00)	0.00 (0.00 – 0.00)
***P* value**	***P*<0.001**	-	-	-	-

a P<0.05

b P<0.05

n, number of habitats; (%), proportions percentage of breeding aquatic habitat types, mean of various vector species is presented alongside the confidence intervals; numbers in parentheses are 95% confidence intervals. P value = significant test at alpha level of 0.05; *An., Anopheles* s.l, sensu lato. The table describes the mean of various malaria vector species per study site and multiple comparisons across the study sites; letters following numbers indicate the results of Tukey-Kramer honestly significant difference tests. Letters in a row show similar significant. -; no analysis of variance since some land use type had no anopheline mosquito count. P values are in bold

**Malaria larval vectors abundance and distribution in land use types across the study areas:** Abundance and distribution of malaria larvae vector species in land use type data is presented in Table 4. High mean abundance of *An. gambiae* s.l was reported in farmland (20.4) relative to pasture land, swamp land, river/stream, artificial forest, bushes and roadsides. The high mean abundance of An. funestus s.l was observed in artificial forest (8.2). High mean abundance of *An. coustani* s.l was observed in artificial forest (4.0). The highest mean abundance of *An. squamosus* was observed on roadsides (1.25).

**Table 4:** Malaria vector abundance and distribution on various land use type across Kakamega county, highlands of western Kenya

Anopheline larval species	Land Use type							*P*-value

	Farmland,	Pasture land,	Swamp land,	River/stream,	Artificial forest,	Bushes,	Roadsides,	
*An.* *gambiae* s.l	20.42 (17.95 – 22.89)	17.65 (11.15 – 24.16)	7.50 (3.08 – 11.92)	7.28 (2.65 – 11.92)	1.44 (0.23 – 3.11)	0.00 (0.00 – 0.00)	3.50 (0.07 – 7.07)	-
*An. funestus* s.l	2.62 (2.04 – 3.19)	2.93 (1.86 – 4.00)	1.74 (0.64 – 2.84)	1.72 (0.25 – 3.19)	8.18 (2.06 – 14.29)	0.00 (0.00 – 0.00)	0.00 (0.00 – 0.00)	-
*An.* *coustani* s.l	1.38 (1.11 – 1.66)	1.46 (0.83 – 2.09)	0.88 (0.10 – 1.86)	0.00 (0.00 – 0.00)	4.03 (1.91 – 6.14)	0.00 (0.00 – 0.00)	1.75 (1.00 – 4.50)	-
*An.* *implexus*	0.05 (0.01 – 0.10)	0.00 (0.00 – 0.00)	0.00 (0.00 – 0.00)	0.00 (0.00 – 0.00)	0.00 (0.00 – 0.00)	0.00 (0.00 – 0.00)	0.00 (0.00 – 0.00)	-
*An.* *squamosus*	0.4 (0.01– 0.08)	0.00 (0.00 – 0.00)	0.00 (0.00 – 0.00)	0.00 (0.00 – 0.00)	0.00 (0.00 – 0.00)	0.00 (0.00 – 0.00)	0.00 (0.00 – 0.00)	-

N, number of habitats; (%), proportions percentage of breeding land use types, mean of various vector species is presented alongside the confidence intervals. *An., Anopheles* s.l, sensu lato; the table describes the mean of various malaria vector species per study site; - no analysis of variance since some land use type had no anopheline mosquito count

## Discussion

Environmental, altitudinal and ecological settings have greatly been associated with density, distribution, occurrence and composition of malaria vectors (3–5,24). Population pressure has led to a shift in land use practices, that is; cultivation of riparian wetland, natural swamps, change in farming practices and massive deforestation (15,17,25,26). Aquatic habitat types and land use changes has demonstrated to influence immature anopheline mosquito productivity and malaria disease transmission in highlands of western Kenya (14–18,27). This study was carried out in Kakamega County within western Kenya highlands to establish abundance and distribution of malaria vector species in various habitat types and land use types.

The malaria vectors identified in this study were An*. gambiae* s.l, *An. funestus* s.l, *An. coustani* s.l, *An. implexus* and *An. Squamosus* occurring in varying proportions between sites, with large changes in abundance and with a possibility of even complete disappearance of *An. implexus* and *An. squamosus* in some study sites. This is consistent with previous studies in western highlands as well as with expectations from this ecological zone as the type of larval sites and land use practices found in western highlands favour development of these species (28). In Malava where *An. gambiae* s.l and *An. coustani* s.l dominated the catch, the frequency of drainage ditches as habitat and farming as a land use type was higher relative to the other sites. Previous studies have found high *An. gambiae* s.l and *An. coustani* s.l larval abundance in agricultural trenches used to drain stagnant water in the farm (29). In Lurambi, where *An. funestus* s.l dominated, majority of habitats were burrow pits, tire tracks, mountain rock pools, and puddles. Naturally, *An. funestus* prefers long lasting water sources like mountain rock pools (30). In Mumias East where *An. implexus* were conspicuously present while *An. squamosus* were present in high density relative to other study sites, majority of the breeding aquatic habitat was tire tracks. During the rainy season water accumulates in tire tracks which serve as larval sites. Such habitats rarely last more than 5 days to enable the larval stages to complete their development (31), this may explain the occasional occurrence *An. implexus* and *An. squamosus* in the study sites. Conjointly, level of permanency may be more influential on the mosquitoes' development at larval stage and thus on species composition. All in all, land use types observed in this area increases habitat creation promoting favorable environment for anopheline species breeding (15,32).

The study characterized larval habitats regarding their physical description and association between the different anopheline species. *An. gambiae* s.l were most abundant in fish ponds which is consistent with previous study in Kisii County, Kenya (33). Fishponds have been recognized as a serious threat for malaria transmission, where the presence of fishponds have been associated with an increase in malaria cases (34). *An. gambiae* s.l, the main malaria vector in western Kenya (35) seems to have adapted well in fishponds despite juvenile predation (36). Burrow pits were the main larval habitat for *An. funestus* and *an. implexus. An. funestus* prefer more semi-permanent large larval habitats like burrow pits which offers refuge during the dry season ensuring persistence of this vector (4,28). *An. coustani* s.l were present more often in drainage ditches which have been identified as larval habitats for malaria vector including *An. coustani* s.l (37). *An. squamosus* was only present in tire tracks which have been identified as the most productive breeding sites in western Kenya (4). There is high diversity of aquatic habitat types and associated malaria vectors in terms breeding preference resulting from habitat hydrological conditions in western Kenya (38). Therefore, treatment of potential aquatic larval habitats should be considered as an additional strategy for malaria vector control.

Land use types perturbs vector composition in terms of anopheline larval distribution and density. *An. gambiae* s.l, *An. implexus* and *An. squamosus* were frequently observed in aquatic habitats located on farmlands. This study partly agrees with previous studies in highlands of western Kenya which demonstrated increased *An. gambiae* s.l and *An. implexus* larval population in farmlands (15,28,39). In the study, drainage ditches were found on farmlands in the four study sites. Agricultural practices such as draining of farmland water using drainage ditches provide suitable breeding grounds for *Anopheles* species (40). *An. funestus* s.l and *An. coustani* s.l were mostly sampled from habitats in artificial forests and this partly agrees with previous studies in Cameroon that reported high densities of *An. funestus* s.l in a forested zone (41). The larval habitats identified in this study site with vegetation cover favours proliferation of *An. funestus* s.l (23). Taken together, land use types influences the growth, survivorship and composition of malaria vectors. Therefore, this can increase vectorial capacity and consequently malaria transmission.

The study did not correlate densities, distribution and composition of various immature malaria vectors in relation to adult malaria vector population. Again since it was a cross-sectional study, the study did not compare the abundance, distribution and occurrence of malaria vectors across all seasons of the year.

In conclusion, the study established that the productivity and composition of anopheles larvae varied across various aquatic habitat types and land use types. Therefore, it is important to potentially target aquatic larval habitats in various land use types in Kenya as an additional strategy for malaria vector control.
